# Prospective impacts of oil spills on floodplain vegetation: Both crude oil and diluted bitumen increase foliar temperatures, senescence and abscission in three cottonwood (*Populus*) species

**DOI:** 10.1371/journal.pone.0230630

**Published:** 2020-03-27

**Authors:** Kayleigh G. Nielson, Samuel G. Woodman, Stewart B. Rood

**Affiliations:** Department of Biological Sciences, University of Lethbridge, Lethbridge, Alberta, Canada; University of Alberta, CANADA

## Abstract

Oil pipelines are vulnerable at river crossings since floods can expose and rupture pipes, releasing oil that floats and coats floodplain vegetation. This study investigated the consequences of oil coatings on leaves of cottonwoods (riparian poplars), the predominant trees in floodplain woodlands around the Northern Hemisphere. The study compared conventional crude oil (CO) versus diluted bitumen (dilbit, DB), heavy oil originating from the Alberta oil sands; with petroleum jelly (PJ) as a reference. The treatments increased leaf surface temperatures (T_leaf_) in narrowleaf and plains cottonwoods (*Populus angustifolia*, *P*. *deltoides*) and balsam poplars (*P*. *balsamifera*) (Control = 21.8°C, PJ = 23.7°C; CO = 26.2°C; DB = 28.1°C; T_air_ = 25°C). The leaf warming followed stomatal occlusion from the foliar coating, which would reduce transpiration and evaporative cooling, combined with increased solar warming with the darker oils. T_leaf_ varied across the three cottonwood species, with cooler, narrow, narrowleaf cottonwood leaves; intermediate plains cottonwood leaves; and warmer, darker, balsam poplar leaves (average T_leaf_: narrowleaf = 23.8°C, plains = 24.3°C, and balsam = 26.7°C), with similar warming in each species following the different treatments. Across species and treatments, T_leaf_ was tightly correlated with foliar condition, which assessed turgor versus wilting of leaf blades and petioles, along with leaf necrosis and senescence (r^2^ = 0.980, narrowleaf; 0.998, plains; 0.852, balsam). This tight association indicates validity of both T_leaf_ and foliar condition as diagnostic measures. Crude oil and dilbit had similar foliar impacts, and for both, leaf abscission occurred within 2 to 3 weeks. Consequently, following an oil spill, remediation should commence quickly but extending vegetation removal beyond a few weeks would have limited benefit since the contaminated leaves would have abscised.

## Introduction

Pipelines provide efficient means of transporting crude oils across North America and worldwide [1]. Occasionally pipelines rupture and result in oil spills, which are followed by oil containment and remediation. Pipelines are particularly vulnerable at river crossings and the risk increases during flood events when swift flows erode the channel bed and banks and flex and rupture the exposed pipe [2,3]. Shut-off valves are commonly installed at river crossing to reduce risk but ruptures persist, including major spills from flood-induced crude oil pipeline ruptures in 2011 along the Yellowstone River, Montana, USA, and in 2012 along the Red Deer River, Alberta, Canada [4].

**Fig 1:**
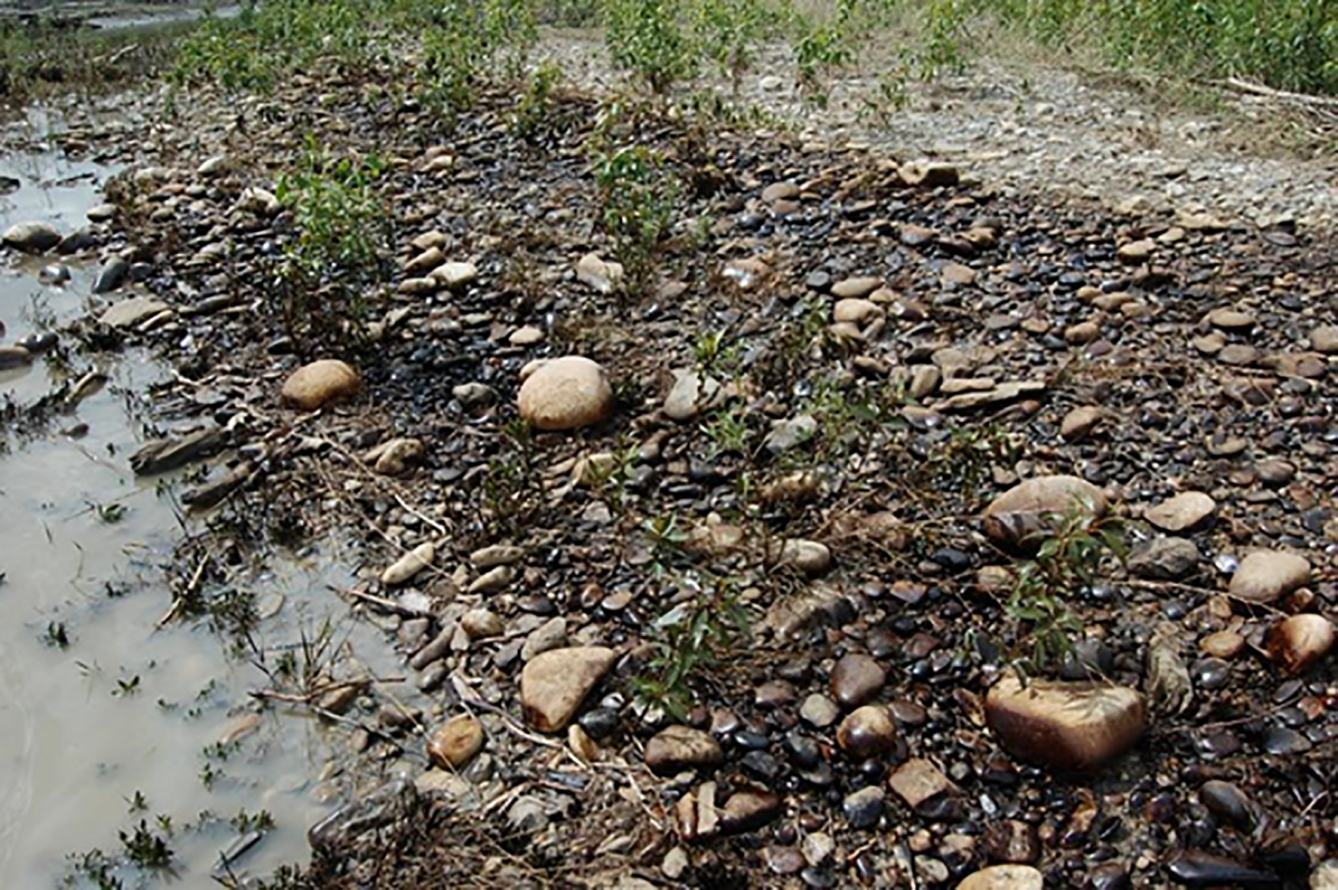
Photograph of crude oil contaminated balsam poplar saplings along the Red Deer, Alberta following the rupture of a crude oil pipeline due to a river flood in June 2012.

When pipelines rupture during flood events, the released oil floats on top of the flood waters that flow over the floodplain [4]. These zones are commonly colonized by riparian vegetation which filter out some of the floating oil, thus coating the stems and leaves, as well as the banks (Fig 1). Along many rivers around the Northern Hemisphere, floodplain woodlands are dominated by cottonwoods, riparian poplar (*Populus*) trees [5], and consequently these species are at greater risk of impact by oil spills. For the clean-up and remediation, some of the most intensive and expensive efforts involve the removal of oil-contaminated riparian vegetation but the nature and timing of the responses to oiling by the cottonwoods or other riparian plants are poorly understood, as are the environmental consequences from the vegetation removal.

Crude oils vary in composition, with lighter crude oils containing smaller and more volatile hydrocarbons that produce a less viscous and free-flowing mixture, while heavy oils have higher proportions of larger hydrocarbons, which increases viscosity, impeding pipeline flow [6]. In North America, the largest oil deposits are in the Athabasca oil sands, where shallow deposits of bitumen, a form of very heavy oil, occur in mixtures with sands and other sediments [7,8,9]. The bitumen is mined, separated from the sands and then diluted with lighter hydrocarbons creating diluted bitumen or ‘dilbit’ for pipeline transport [9,10].

There are proposals for additional pipelines to transport dilbit, including Keystone XL in the United States, and the Trans Mountain Pipeline Expansion in Canada [11,12]. A focus of concern is the environmental hazard from pipeline failures and while there is limited understanding of the environmental impacts of crude oil spills on floodplain ecosystems, there is even less knowledge about the hazard from dilbit. Some scientific studies followed a dilbit spill into the Kalamazoo River in Michigan in 2010 [13] and there have been comparisons of the effects of crude oil versus dilbit on some fish and aquatic organisms [14,15] but no prior comparison of the consequences for riparian vegetation.

There have been a few studies of controlled crude oil applications to upland forests [16,17,18,19,20] and a number of studies of marsh plants, including some following oil spills in the Gulf of Mexico [21,22,23,24]. From these studies, a primary effect from the oil coating of leaves is stomatal occlusion that blocks gas exchange [22,25], the counter-current influx of CO_2_ for photosynthesis and efflux of water vapor with transpiration. This suffocates the leaves, leading to senescence and abscission. The blockage of foliar transpiration also reduces evaporative cooling, which leads to leaf warming that can be detected with infra-red thermal imaging [26,27,28,29].

Following from the foliar responses in marsh plant it seemed likely that similar impacts would follow oil contamination of cottonwood leaves. This would lead to stomatal occlusion that would block transpiration and result in leaf warming that could be quantified with thermal imaging. If the blockage persisted, the leaves would senesce and abscise. The relative severity of contamination with crude oil or dilbit could subsequently by compared by analyzing foliar morphology and physiology, including leaf warming and the subsequent decline in foliar condition.

This study was undertaken to investigate prospective environmental impacts from oil spills that contaminate floodplain vegetation, with three objectives:

1. To assess stomatal blockage, leaf temperatures and foliar condition following contamination with conventional crude oil versus dilbit;
2. To compare the responses across three cottonwood species to investigate interspecific variation; and
3. To analyze the timing of foliar responses, which would relate to the urgency and scheduling of remediation following oil spills.

## Materials and methods

### Study site and populus species

The study was conducted in the University of Lethbridge nursery in Alberta, Canada (49°41’ N, 112°51’ W, 917 m asl), where cottonwoods collected from regional riparian zones were clonally propagated in 1983 [30]. Three native riparian poplar species were compared: narrowleaf cottonwood, *Populus angustifolia* James, and balsam poplar, *P*. *balsamifera* L., of section *Tacamahaca*; and the plains or prairie cottonwood, *P*. *deltoides* Bartr. ex Marsh. ssp. *monilifera* (Ait.) Eckenw., of section *Aigeiros*; these three will be collectively referred to as ‘cottonwoods’. Trees at the southern margin of the nursery were selected since they were exposed to mid-day sunshine, similar to cottonwood saplings in lower floodplain zones most vulnerable to oil spills (Fig 1). The same plains cottonwood and balsam poplar trees were used across the different experiments, but two narrowleaf trees were used due to a limited number of available branches for treatment. The study site was adjacent to plots for analyses of narrowleaf cottonwoods by Kaluthota et al. [31], and that report provides further information about foliar physiology and environmental conditions at the study site.

### Petroleum substances

The experimental study involved four comparative treatments, including a control (C) with no substance applied. Petroleum jelly (PJ; Vaseline®, Unilever Canada, Toronto, ON) provided a reference substance. It is relatively clear and has long been used experimentally to coat plant leaves to block foliar gas exchange, including transpiration [32,33]. The crude oil (CO) was a light, sweet (low hydrogen sulfide) type [29] extracted from a well near Barons, Alberta in 2013 (Penn West Exploration). The diluted bitumen (dilbit, DB; lot ‘CLK#19’ from Natural Resources Canada, Devon, AB) was extracted from a site near Cold Lake, Alberta and diluted approximately 30% with natural gas condensate [9,34].

### Experimental treatments

Experiments commenced June 17, 2013 with crude oil and reference treatments applied to leaves of the three cottonwood species. The dilbit was obtained later, with that treatment starting August 1, 2013, after stem growth cessation [31]. The experimental study was repeated two years later starting on June 16, 2015, with all treatments applied at the same time. The results were similar from the 2013 and 2015 experiments, and we primarily present results from the 2015 experiment that included the concurrent application of the four treatments.

**Fig 2:**
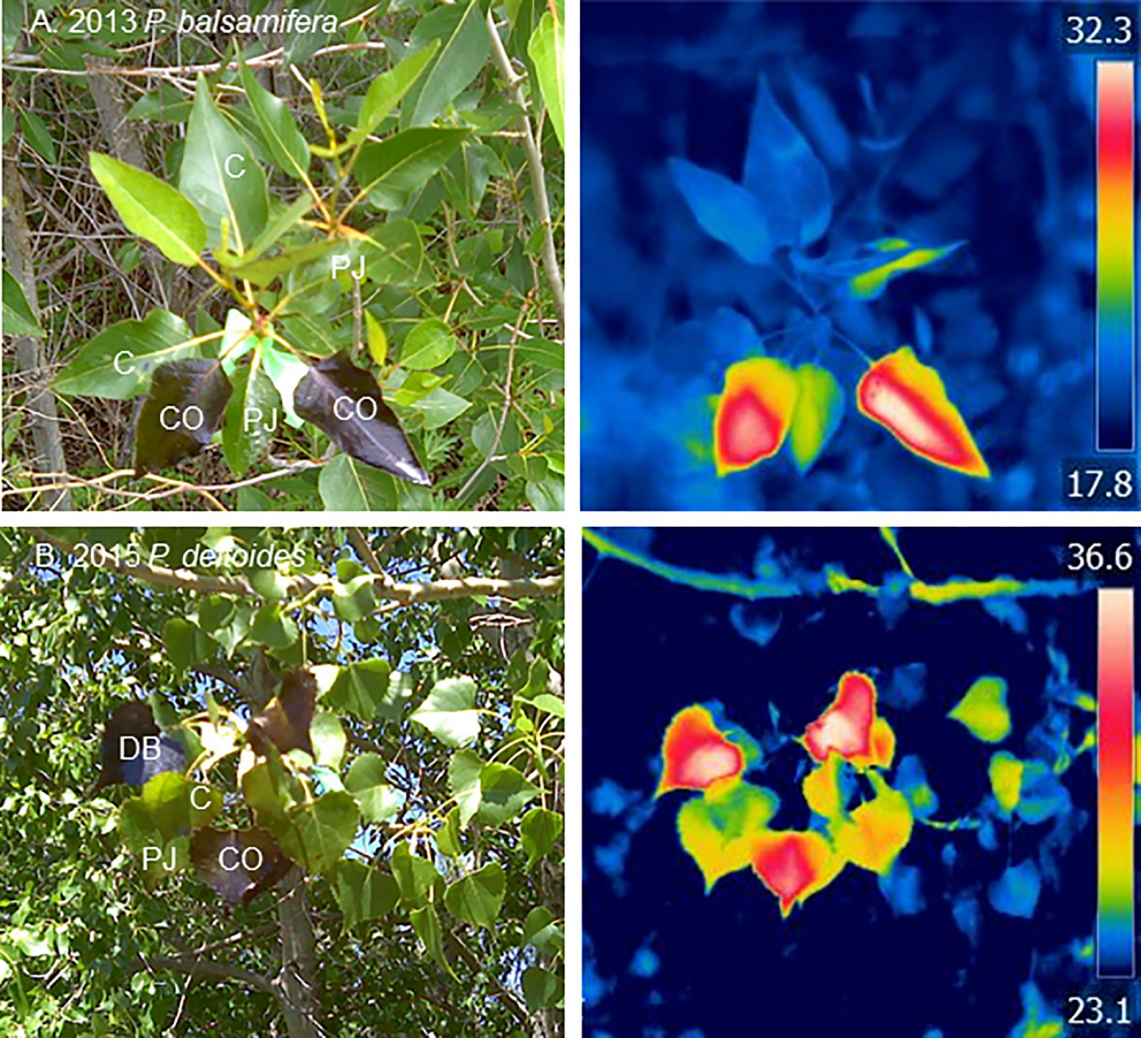
Photographs and corresponding infra-red thermal images of leaves of cottonwood species *P*. *balsamifera* (A., top, 2013 experiment, T_air_ ~ 20°C) and *P*. *deltoides* (B., bottom, 2015 experiment, T_air_ ~ 25°C), following applications of petroleum substances (C = control, PJ = petroleum jelly, CO = crude oil, DB = diluted bitumen).

For each experiment, three uniform and apparently healthy long-shoot branches 1 to 1.5 m above ground were selected from each study tree, with different branches selected for each experiment. On each branch, two healthy, fully expanded leaves were randomly assigned for each of the three (2013) or four (2015) treatments: C, PJ, CO and DB (Fig 2). This provided a factorial study design with 3 branches × 2 leaves (6 replicates) for each of 9 (3 treatments × 3 species, 2013) or 12 conditions (4 treatments × 3 species, 2015) resulting in 54 or 72 study leaves that were each assessed on multiple dates. A sponge brush was used to apply an even coating of each treatment to the entire leaf blade to both the adaxial (upper) and abaxial (lower) surfaces; both surfaces of a leaf would be coated during a floodplain oil spill and cottonwood leaves are amphistomatous [35].

### Assessment of foliar condition

Foliar condition, a visual assessment of health of each of the 54 (2013) or 72 (2015) leaves was assessed by the same observer on the treatment day, the following day and then at two to four-day intervals, around mid-day (11:00 to 15:00), the period with maximal transpiration [36]. Foliar condition was rated on a scale from one to five, with five indicating an apparently healthy, dark green, turgid leaf blade and petiole [37,38]. The score declined to one, representing a wilted, flaccid leaf with extensive senescence and necrosis, yellowing and browning. Zero was assigned with leaf abscission, and the date recorded. The numbers of study leaves declined after about ten days as leaves died, and for each experiment the foliar assessments and other measurements were undertaken until all oiled leaves had abscised, after about four weeks.

### Leaf surface temperatures—infra-red thermal imaging

To assess foliar warming, thermal images along with matching digital images, were taken using a FLIR B300 infra-red camera (FLIR Systems Inc., Wilsonville, OR, USA). Experimental leaves were photographed on the day after treatment and then at four or six day intervals until all treated leaves had abscised. Each image was calibrated to the ambient conditions at the time of capture according to FLIR QuickReport 1.2 SP2 (FLIR Systems Inc. 2009). The imaging distance was approximately 1 m and calibration parameters included: the leaf emissivity (0.965), the measured reflected apparent temperature and the ambient air temperature (T_air_), and relative humidity measured at the Environment Canada weather station at the Lethbridge airport, at similar altitude 6.5 km away. Thermal images were analyzed using the FLIR spot tool to measure the surface temperature at about 15 approximately evenly-spaced points on each treated leaf and these were averaged to provide the mean surface temperature for that leaf (T_leaf_) on that date.

Weather varied somewhat over the study intervals and for the concurrent 2015 study, more uniform cloudless conditions with bright sun occurred on June 16, 22 and 30. There was substantial leaf abscission for the oil treatments by June 30 and consequently the statistical analyses assessed T_leaf_ from June 16 and 22, 2015. On those days, the maximum air temperatures at the nearby Lethbridge Airport were 25.9°C and 26.1°C, respectively (Environment Canada; climate.weather.gc.ca), and air temperatures during the afternoon measurements (T_air_) were approximately 1°C cooler (25°C). This measurement interval was fairly dry with light showers providing 0.3 and 1.5 mm of precipitation in the three days preceding June 16 and June 22, respectively. The June 2015 total precipitation of 13.4 mm was well below the average of 44.2 mm, and afternoon winds were typically moderate through June, with daily peak gusts averaging 38 km/h. June daylengths range from 16:01 to 16:19 (hr:min) and on June 16 and 22 the total incoming radiation was 33.1 and 31.6 MJ/m^2^, respectively (Alberta Climate Information Service).

### Leaf surface imaging–environmental scanning electron microscopy

To visualize the leaf surfaces and stomata following the different treatments, fresh leaves from each species and treatment condition were collected. Small discs were punched between the midrib and leaf margin and mounted on aluminum SEM stubs using double-sided adhesive tape, with either the adaxial (top) or abaxial surfaces displayed. Surface imaging utilized a Hitachi High-Technologies TM-1000 environmental scanning electron microscope (ESEM; Hitachi High-Technologies Europe, Krefeld, Germany). This instrument has differential vacuum pumping and other modifications to enable imaging of fresh specimens. Viewing was generally at 300x magnification and multiple images were saved from each sample for stomatal observations [39].

### Statistical analyses

The study design included three factors, with (1) species and (2) treatment as the focus. The leaves were assessed on (3) different dates, and were grouped by branches, which provided a possible secondary influence. The outcomes were very consistent across the three experiments over two years, and we emphasize the results from the 2015 experiment with concurrent treatments including CO and DB. For that experiment, the final analysis for T_leaf_ was with a linear mixed model analysis in SPSS (IBM, NY) with *species*, *treatment* and *species* × *treatment* specified as fixed effects, *date* included as a repeated effect, and *branch* × *leaf* as the subject variable. Subsequent marginal means were compared with Least Significant Differences (LSD) with Bonferroni adjustments, for paired comparisons across the three species, the four treatments, and then for the different species and treatment combinations. Correspondences between foliar condition and T_leaf_ were explored with linear regression with the different treatments for each cottonwood species with SPSS.

## Results

### Leaf surface temperatures

**Fig 3:**
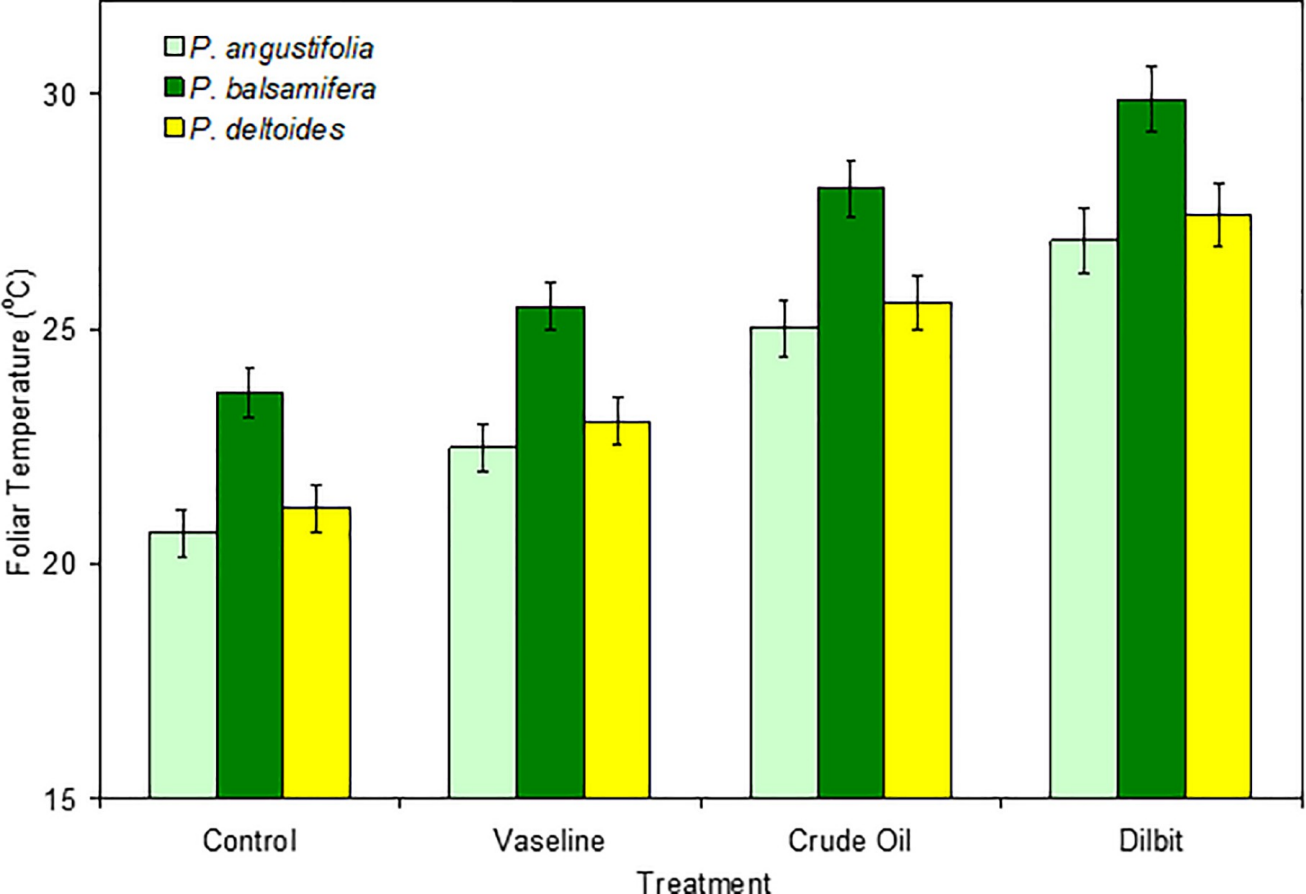
Foliar (leaf) temperatures (mean ± SE) of three cottonwood (*Populus*) species after applications of petroleum substances, June 16 and 22, 2015 (dilbit = diluted bitumen; T_air_ ~ 25°C).

Following foliar application of the petroleum substances, the first measurable change was an increase in leaf surface temperatures (Fig 2). The coated leaves were warmer by the first infra-red measurements on the day following the experimental treatments, and the temperature patterns were fairly similar over the study intervals of two to three weeks. With averaging across leaves, species and dates, the petroleum jelly (PJ), crude oil (CO) and dilbit (DB) coated leaves averaged 1.83°C, 4.37°C and 6.25°C warmer than the untreated controls (C) (Fig 3).

The mixed model analysis of variance revealed highly significant effects of species and of treatment on T_leaf_ (Table 1; *p* < 0.001). There was no significant interaction between species × treatment (Table 1) and thus the T_leaf_ were similarly increased with the different treatments across the three species (Fig 3). The post-hoc pairings indicated that T_leaf_ differed between all three species (narrowleaf vs. plains: *p* = 0.022; other pairings: *p* = 0.001).

**Table 1:** Effects from the linear mixed model analysis of leaf temperatures (T_leaf_) from 6 leaves (2 leaves × 3 branches) of each of 3 cottonwood species, and 4 oiling treatments (3 species × 4 treatments = 12 experimental conditions), assessed by infra-red thermography on 2 uniformly sunny dates (repeated measures), June 16 and 22, 2015 (df = degrees of freedom).

Source	Numerator df	Denominator df	F	Significance
Intercept	1	110	21028	.000
Species	2	110	21.1	.000
Treatment	3	69.4	27.8	.000
Species × Treatment	6	66.7	.593	.753
Date	1	103	116	.000

For the post-hoc comparisons between treatments, the crude oil (CO) and dilbit (DB) treated leaves were significantly different than the control (C) or petroleum jelly (PJ) treated leaves (*p*<0.01). Across the three species, there appeared to be a trend with higher T_leaf_ in the DB-treated than the CO-treated leaves (Fig 3) but these did not significantly differ (*p* = 0.119) in the 2015 study. Apparently increased warming with DB versus CO was also observed in the 2013 experiments (C = 21.82°C ± 0.42°C (SE); CO = 26.16°C ± 0.54°C; DB = 28.10°C ± 0.65°C) and while this difference would have been statistically significant (LSD test, *p* = 0.020), the two treatments were imposed at different times and are not directly comparable. There was significant warming with PJ in 2013 (analysis with treatments C and PJ: species *p* = 0.000; treatment *p* = 0.000; species x treatment *p* = 0.987) but the apparent warming with PJ was not statistically significant in the 2015 experiment (*p* = 0.169).

### Leaf morphology

**Fig 4:**
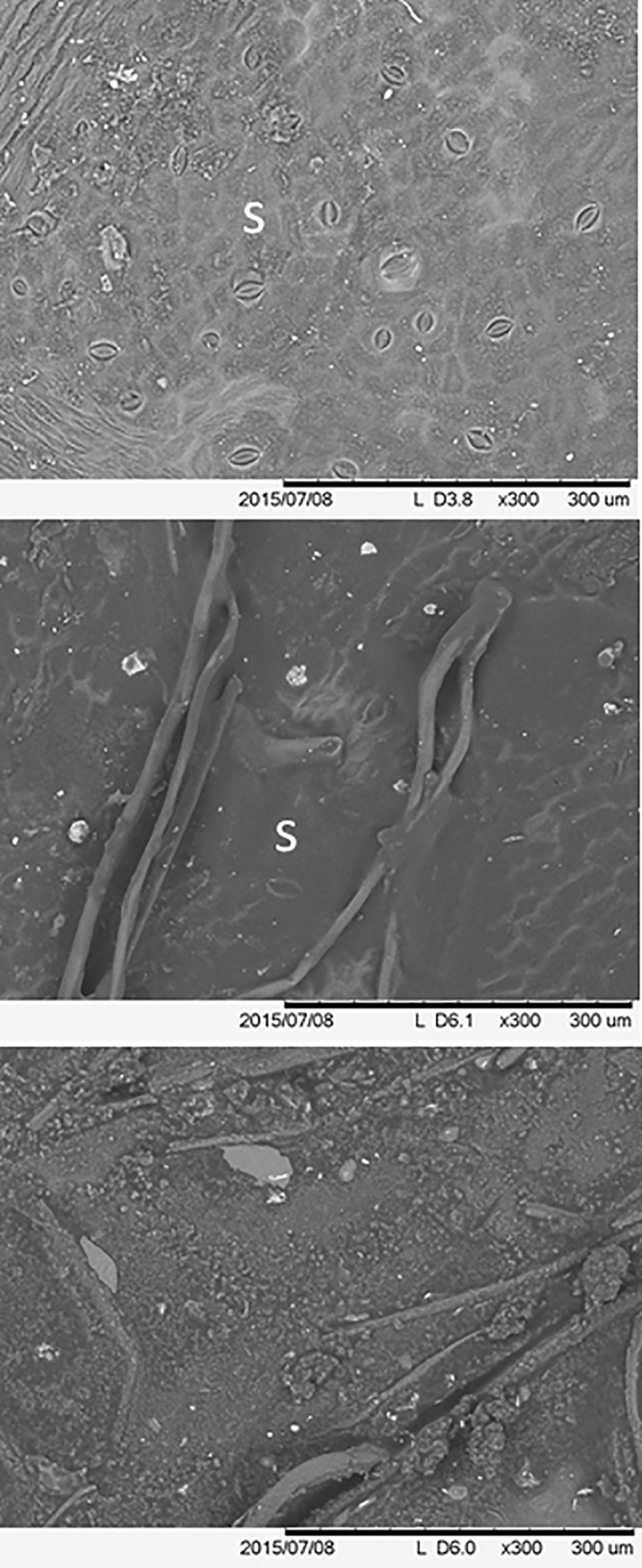
Scanning electron photomicrographs of the adaxial (upper) surfaces of *P*. *deltoides* leaves, with control leaves (top) and those coated with crude oil (middle) or diluted bitumen (dilbit, bottom) s = stomata.

The environmental scanning electron microscopy (ESEM) displayed the leaf surface coatings with the petroleum substances (Fig 4). The untreated, control leaves displayed natural surfaces with exposed epidermal cells and the regulatory guard cells that flank the stomata, the surface pores that permit transpiration. In contrast, with all three petroleum substance treatments, the leaf surfaces were covered with the coatings and the stomata were occluded. There were also apparent strands that formed with the evaporation of the lighter hydrocarbons with the CO and DB treatments (Fig 4). Particles in the coverings would originate from the dilbit, along with dusts and other airborne particles that adhered to the coatings when they were initially sticky.

### Foliar condition

**Fig 5:**
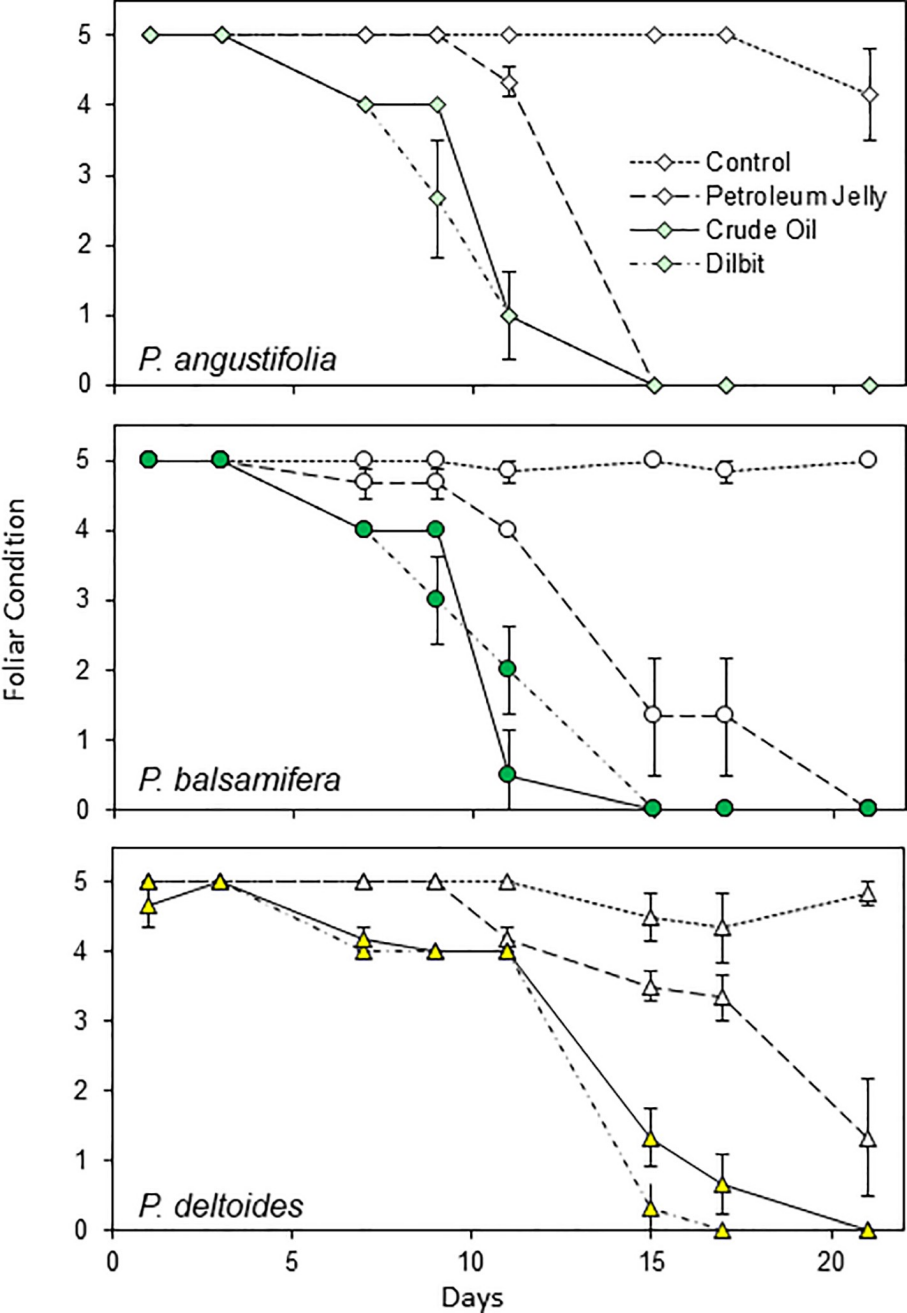
Decline in foliar condition over three weeks, following the applications of petroleum substances (dilbit = diluted bitumen) to three cottonwood (*Populus*) species in the 2015 experiment. Mean ± SE are plotted, and symbols are colored for CO and DB, matching species colors in Fig 3.

**Fig 6:**
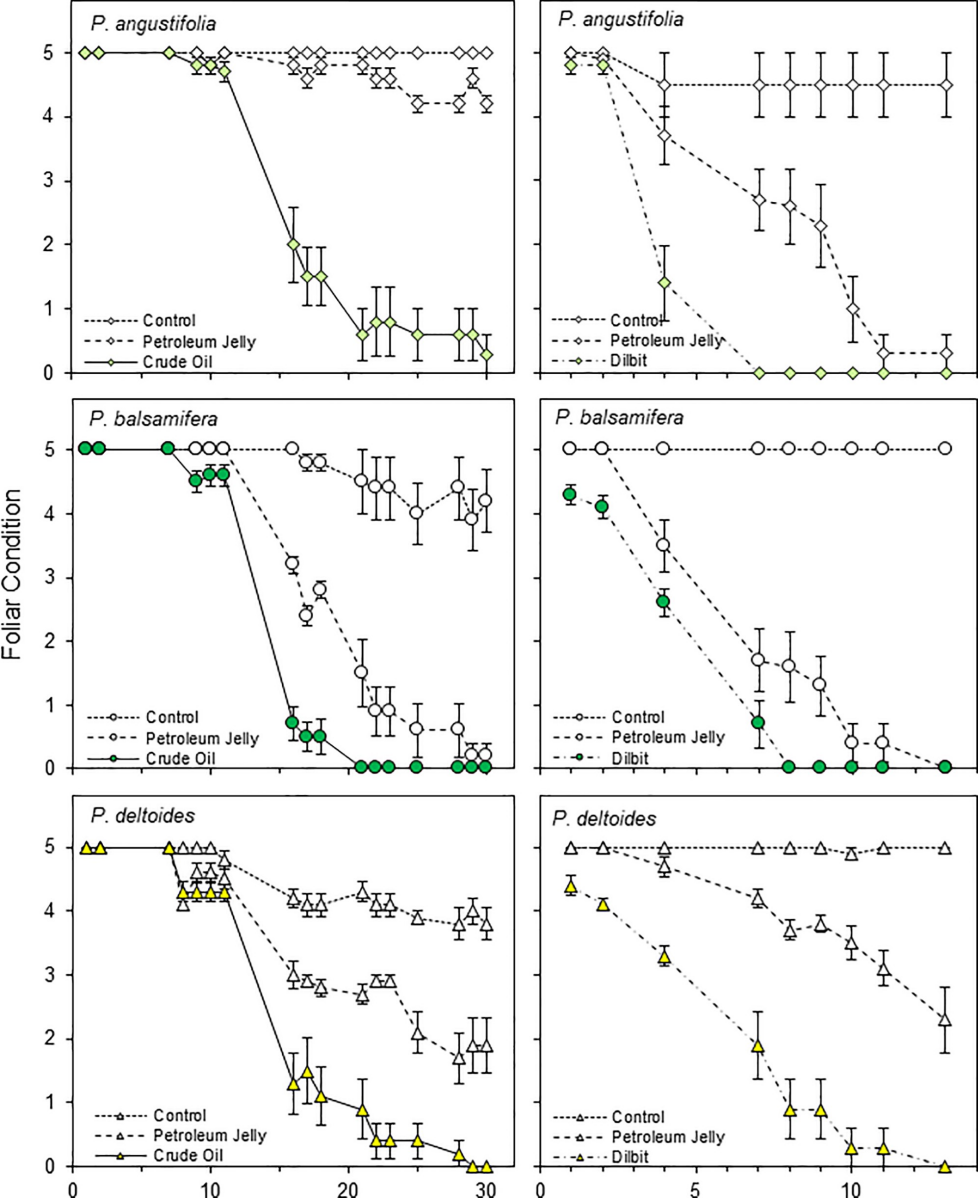
Decline in foliar condition following the applications of petroleum substances to three cottonwood (*Populus*) species in the 2013 experiments. The left column represents the June experiment with crude oil (CO), and the right column represents the August experiment with diluted bitumen (dilbit, DB), and note the different x-axis time scaling. Both experiments included the reference substance, petroleum jelly, and uncoated controls, with mean ± SE plotted. Symbols are colored for CO or DB, matching species colors in Fig 3.

Along with leaf T the second primary measure was foliar condition, which progressively declined after about five days following treatments with the petroleum substances. As displayed in Fig 5 for the 2015 study with concurrent treatments, and in Fig 6 for the 2013 experiments with sequential treatments, the treatments provided generally similar declines in foliar condition for the three cottonwood species. An exception was for the June 2013 application of PJ to the narrowleaf cottonwoods, which had relatively slight impact on foliar condition (Fig 6, upper left). Conversely, the repeat treatment in June 2015 provided similar foliar decline in narrowleaf cottonwood as for the other two species (Fig 5). For all three experiments, the plains cottonwood leaves responded slightly more slowly than the narrowleaf cottonwood and balsam poplar leaves.

Across the substances, there were generally similar patterns of foliar decline for the CO and DB (Fig 5). PJ was also lethal but with a more gradual response. PJ provided a common reference for the two experiments in 2013 and revealed that the leaf decline was faster in late summer than in early summer.

**Fig 7:**
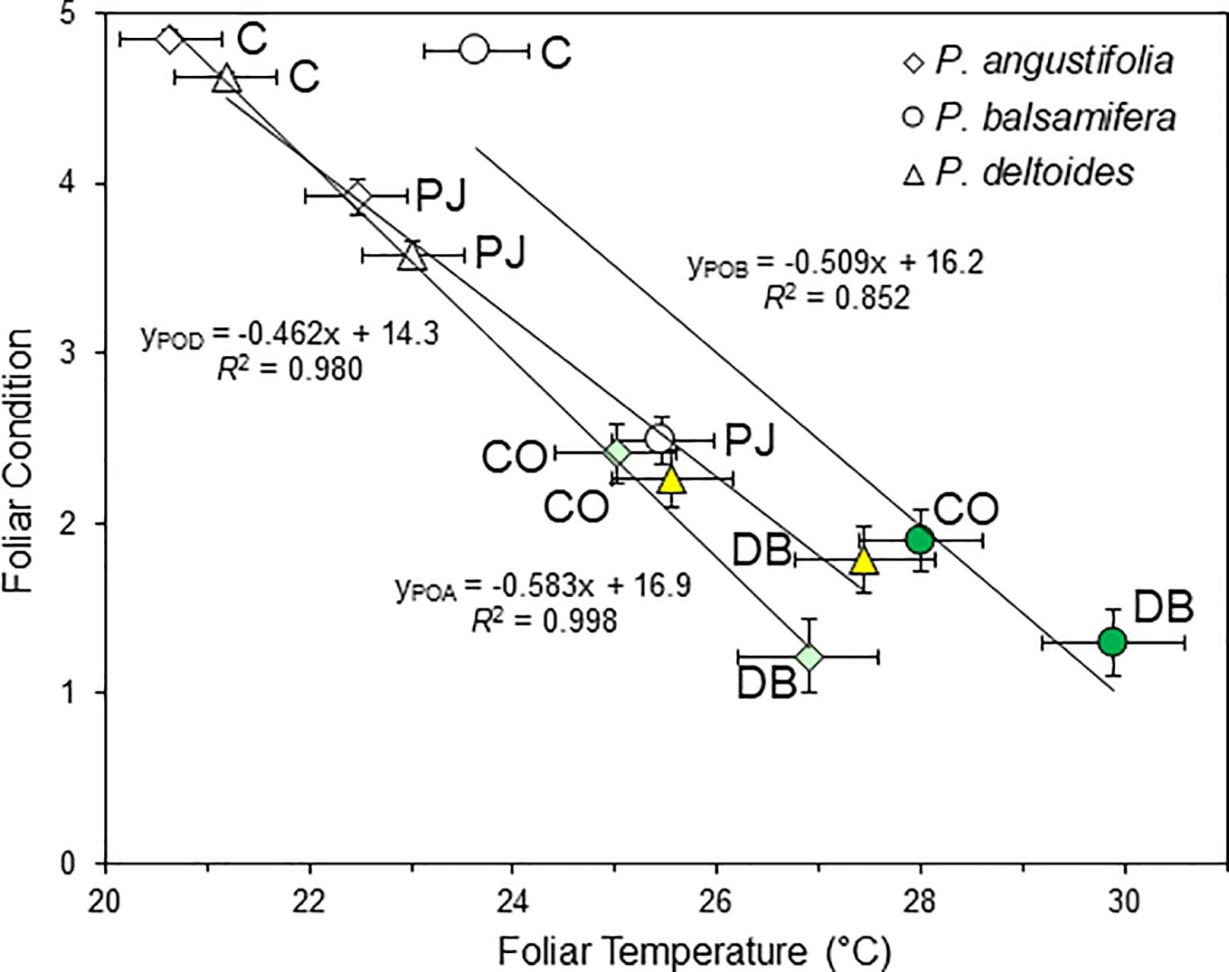
Correspondence between foliar condition and leaf surface temperatures of three riparian cottonwood (*Populus*) species in the 2015 experiment. Mean ± SE are provided for both axes, but vertical standard error bars are smaller than the symbols for controls (C = control, PJ = petroleum jelly, CO = crude oil, DB = diluted bitumen; all regressions, *p* < 0.01). Comparisons represent data collected June 16 and 22, 2015. Symbols are colored for CO and DB, matching species colors in Fig 3.

The foliar condition assessments were very tightly correlated with the instrumentally measured leaf temperatures (Fig 7). There was nearly complete linear correspondence for the plains and narrowleaf cottonwoods and some deviation with the PJ treatment to the balsam poplar leaves (Fig 7). The tight correspondence between the two measures validates the two independent methods of foliar assessment.

## Discussion

### Foliar responses to petroleum substances

The study was undertaken with three objectives, with the first to characterize the foliar morphological and physiological responses following oil treatments. The first observed response was the increase in leaf surface temperature. Relative to mechanism, the coatings with each of the petroleum substances resulted in stomatal occlusion, and this would block transpiration and reduce the associated evaporative cooling [16,28]. Prior studies have demonstrated the blockage of transpiration with petroleum jelly (PJ) [26,33,40], and our study indicates a similar response to foliar coatings of crude oil (CO) or dilbit (DB). Leaf surface temperatures were higher following CO or DJ treatment than PJ (Figs 3 and 7) and while all three substances would block transpiration, the darker CO or DB coatings would absorb more solar radiation due to reduced albedo and this would increase the foliar warming [32].

In addition to blocking transpirational water efflux, the stomatal occlusion would exclude the CO_2_ uptake for photosynthesis. This would starve the leaves, contributing to the observed decline, senescence and mortality [16,22,25]. Darkening the leaves with CO or DB would also reduce light transmission, further reducing photosynthesis and inducing senescence [41].

The foliar warming could also contribute to metabolic stress [42]. Thus, the foliar decline and mortality would reflect additive or possibly compounding influences from (1) carbon and energy starvation due to the blockage of carbon dioxide uptake and photosynthesis, (2) metabolic stress from warming temperatures and (3) reduced water and nutrient uptake with the blockage of transpiration. All three petroleum substances would reduce photosynthesis and transpiration, while the increased warming with CO or DB could increase the metabolic stress, accelerating the foliar decline and abscision, as was observed (Figs 5 and 6).

### Diluted bitumen versus crude oil

While previous studies have investigated CO contamination of upland vegetation or marsh plants, a novel focus of the study was to assess the foliar responses to DB. The thermal imaging indicated slightly greater warming following the DB treatments and this probably partly reflected thicker and darker coatings since the DB was more viscous compared to CO. We expected that the responses to DB or CO could be generally similar, and for both, thicker coatings would increase the response severity. The foliar condition decline rates were similar for DB versus CO for all three cottonwood species (Fig 5), and this would indicate generally similar impact from these two substances.

These results combined with prior reports suggest that the lethal stress was primarily physical and physiological, rather than from toxicity from the petroleum substances [16,17,18]. Supporting this interpretation, bitumen mulch or emulsion has been used as a soil amendment for revegetation of sand dunes, gravel patches and other sites with challenging substrates, suggesting limited toxicity [43,44]. Some constituents in DB are shared with asphalt, which is a primary material for paving roadways, and there is generally little concern for the toxicity to adjacent vegetation [45]. DB and CO mixtures do contain several toxic components, including benzene, toluene, ethyl-benzene, and xylene (‘BTEX’, [46]) but these are highly volatile and would readily evaporate following a spill after a pipeline rupture, reducing the chemical hazard to floodplain vegetation.

### Sensitivity across populus species

Our second objective was to compare responses across three cottonwood species from two *Populus* sections. The three generally responded similarly to the three petroleum substances with similar morphological consequences and physiological responses. There were some differences, including warmer surface temperatures of the balsam poplar leaves (Fig 3), which were naturally darker. In control or treated leaves, narrowleaf cottonwood (*P*. *angustifolia*) leaves were the coolest and these had narrow leaves that could increase the boundary layer conductance and consequently reduce warming [29]. The longer, flexible petioles of the plains cottonwood (*P*. *deltoides*) would increase leaf fluttering and air exchange [47], and also provide more vertical leaf positions (Fig 2), which could also reduce warming by limiting direct exposure to sunlight. The differences in leaf temperatures could also reflect differences in stomatal densities and distributions across the three cottonwood species, since these would influence transpiration patterns [35].

For the response timings, the two section *Tacamahaca* species, *P*. *angustifolia* and *P*. *balsamifera* displayed similar time-courses, while foliar mortality was slightly delayed in *P*. *deltoides* of section *Aigeiros* (Figs 5 and 6). Stomata are more uniformly distributed between the adaxial and abaxial surfaces in *P*. *deltoides* [35], and the leaf flutter could also provide some benefit for gas exchange [47]. Conversely, the differences across the species were minor and we conclude that different riparian *Populus* species would probably display generally similar responses to oiling following pipeline ruptures with river floods.

There was a difference in the response of the narrowleaf cottonwood to the reference substance of PJ in the June 2013 experiment (Fig 6, upper left). In contrast to the responsiveness of the other two poplars, this treatment combination produced only slight decline in foliar condition over the four-week study. The narrowleaf cottonwood often occurs in low elevations along rivers and may be more flood tolerant than other riparian poplars [48]. Flooding imposes root anoxia, which impedes water uptake and induces drought stress and stomatal closure [48]. Consequently, narrowleaf cottonwood may be more tolerant of a temporary block to foliar gas exchange. Conversely, the response of narrowleaf cottonwood to the PJ coating was more similar to that of the other two poplar species in the August 2013 and June 2015 experiments, suggesting generally similar responses across the three poplar species.

While the different species displayed generally similar responses to oiling, the likelihood of contamination could vary across riparian tree and shrub species. The elevational distribution of riparian poplars varies somewhat, with the narrowleaf cottonwood often occurring in lower positions closer to the river, while the plains cottonwoods can occur further away and at higher elevations above the stream [5,49]. This elevational segregation could increase the likelihood of contamination of narrowleaf cottonwoods.

While this study investigated riparian poplars, riparian willows are related members of the Salicaceae and occur in floodplain habitat. The sandbar or coyote willow, *Salix exigua*, is generally the lowest elevation woody plant along North American streams [50] and would be especially prone to oil contamination following pipeline ruptures in river crossings. This is a narrowleaf willow and some of the differences in form and vulnerability displayed between the narrowleaf cottonwood and the broad-leaved cottonwoods might be amplified in this willow species [5].

### Response timing

Our final study objective was to assess the response timing, and this would relate to the urgency and scheduling of vegetation removal following an oil spill with a river flood. Increased leaf surface temperatures were observed within a day following the experimental treatments and the decline in foliar condition was observable within about a week (Figs 5 and 6). The foliar decline was progressive, with mortality and abscission by two to three weeks. Additionally, the leaves of shrubs and trees are elevated and more exposed to wind and sun than some understory plants. Consequently, the volatile hydrocarbons would evaporate within intervals of hours to days, and continuing oxidation would produce chalky stem residues that would no longer be transferable to wildlife [4,51]. With this response timing, it could be appropriate to respond quickly to contain the spill, remove any pooled oil, undertake measures to exclude wildlife, and remove heavily contaminated vegetation. After leaf abscission in about three or four weeks, further vegetation removal would be less useful, and extending clean-up activities could increase the risk of invasion or expansion of weeds and other non-native plants, imposing an unfavorable consequence of prolonged remediation [52].

### Study methodology

The study involved the application of the three petroleum-based substances with paint brushes, which was efficient but the application thicknesses may have varied somewhat. A future application could involve dipping the leaves, branches and even whole potted saplings into the oil [25]. This might provide a more uniform application that better simulates the contamination with floating oil following a flood-induced pipeline rupture. Repetitive dipping could also enable quantitative treatments.

## Conclusion

In this study, we applied two petroleum substances that are of current concern relative to spills from pipeline ruptures at river crossings. We found that crude oil and diluted bitumen (dilbit) had relatively similar impacts following foliar application to three cottonwood species, leading to foliar decline and abscission within about three weeks. The substances acted by coating the leaf surfaces and occluding stomata, which would block gas exchange for transpiration and photosynthesis. The dark coatings would also reduce light for photosynthesis and increase the absorption of solar radiation. This, combined with loss of evaporative cooling with transpiration, resulted in warmer leaf surfaces and we conclude that thermal imaging should provide an effective and quantitative method of assessing contamination of floodplain vegetation following an oil spill from a pipeline failure. Finally, as a management recommendation, due to the abscission of oil coated leaves, we recommend that remedial vegetation removal cease after about four weeks. The further environmental impact would be limited, while the remediation activities could extend the damage to riparian ecosystems.
